# VSEARCH: a versatile open source tool for metagenomics

**DOI:** 10.7717/peerj.2584

**Published:** 2016-10-18

**Authors:** Torbjørn Rognes, Tomáš Flouri, Ben Nichols, Christopher Quince, Frédéric Mahé

**Affiliations:** 1 Department of Informatics, University of Oslo, Oslo, Norway; 2 Department of Microbiology, Oslo University Hospital, Oslo, Norway; 3 Heidelberg Institute for Theoretical Studies, Heidelberg, Germany; 4 Institute for Theoretical Informatics, Karlsruhe Institute of Technology, Karlsruhe, Germany; 5 School of Engineering, University of Glasgow, Glasgow, United Kingdom; 6 Warwick Medical School, University of Warwick, Coventry, United Kingdom; 7 Department of Ecology, University of Kaiserslautern, Kaiserslautern, Germany; 8 UMR LSTM, CIRAD, Montpellier, France

**Keywords:** Clustering, Chimera detection, Searching, Masking, Shuffling, Parallellization, Metagenomics, Alignment, Sequences, Dereplication

## Abstract

**Background:**

VSEARCH is an open source and free of charge multithreaded 64-bit tool for processing and preparing metagenomics, genomics and population genomics nucleotide sequence data. It is designed as an alternative to the widely used USEARCH tool (Edgar, 2010) for which the source code is not publicly available, algorithm details are only rudimentarily described, and only a memory-confined 32-bit version is freely available for academic use.

**Methods:**

When searching nucleotide sequences, VSEARCH uses a fast heuristic based on words shared by the query and target sequences in order to quickly identify similar sequences, a similar strategy is probably used in USEARCH. VSEARCH then performs optimal global sequence alignment of the query against potential target sequences, using full dynamic programming instead of the seed-and-extend heuristic used by USEARCH. Pairwise alignments are computed in parallel using vectorisation and multiple threads.

**Results:**

VSEARCH includes most commands for analysing nucleotide sequences available in USEARCH version 7 and several of those available in USEARCH version 8, including searching (exact or based on global alignment), clustering by similarity (using length pre-sorting, abundance pre-sorting or a user-defined order), chimera detection (reference-based or *de novo*), dereplication (full length or prefix), pairwise alignment, reverse complementation, sorting, and subsampling. VSEARCH also includes commands for FASTQ file processing, i.e., format detection, filtering, read quality statistics, and merging of paired reads. Furthermore, VSEARCH extends functionality with several new commands and improvements, including shuffling, rereplication, masking of low-complexity sequences with the well-known DUST algorithm, a choice among different similarity definitions, and FASTQ file format conversion. VSEARCH is here shown to be more accurate than USEARCH when performing searching, clustering, chimera detection and subsampling, while on a par with USEARCH for paired-ends read merging. VSEARCH is slower than USEARCH when performing clustering and chimera detection, but significantly faster when performing paired-end reads merging and dereplication. VSEARCH is available at https://github.com/torognes/vsearch under either the BSD 2-clause license or the GNU General Public License version 3.0.

**Discussion:**

VSEARCH has been shown to be a fast, accurate and full-fledged alternative to USEARCH. A free and open-source versatile tool for sequence analysis is now available to the metagenomics community.

## Introduction

Rockström et al. (2009) and Steffen et al. (2015) presented biodiversity loss as a major threat for the short-term survival of humanity. Recent progress in sequencing technologies have made possible large scale studies of environmental genetic diversity, from deep sea hydrothermal vents to Antarctic lakes (Karsenti et al., 2011), and from tropical forests to Siberian steppes (Gilbert, Jansson & Knight, 2014). Recent clinical studies have shown the importance of the microbiomes of our bodies and daily environments for human health (Human Microbiome Project Consortium, 2012). Usually focusing on universal markers (e.g., 16S rRNA, ITS, COI), these targeted metagenomics studies produce many millions of sequences, and require open-source, fast and memory efficient tools to facilitate their ecological interpretation.

Several pipelines have been developed for microbiome analysis, among which mothur (Schloss et al., 2009), QIIME (Caporaso et al., 2010), and UPARSE (Edgar, 2013) are the most popular. QIIME and UPARSE are both based on USEARCH (Edgar, 2010), a set of tools designed and implemented by Robert C. Edgar, and available at http://drive5.com/usearch/. USEARCH offers a great number of commands and options to manipulate and analyse FASTQ and FASTA files. However, the source code of USEARCH is not publicly available, algorithm details are only rudimentarily described, and only a memory-confined 32-bit version is freely available for academic use.

We believe that the existence of open-source solutions is beneficial for end-users and can invigorate research activities. For this reason, we have undertaken to offer a high quality open-source alternative to USEARCH, freely available to users without any memory limitation. VSEARCH includes most of the USEARCH functions in common use, and further development may add additional features. Here we describe the details of the VSEARCH implementation. To assess its performance in terms of speed and quality of results, we have evaluated some of the most important functions (searching, clustering, chimera detection and subsampling) and compared them to USEARCH. We find that VSEARCH delivers results that are better or on a par with USEARCH results.

## Materials and Methods

### Algorithms and implementation

Below is a brief description of the most important functions of VSEARCH and details of their implementation. VSEARCH command line options are shown in italics, and should be preceded by a single (-) or double dash (-TI:\,-) when used.

#### Reading FASTA and FASTQ files

Most VSEARCH commands read files in FASTA or FASTQ format. The parser for FASTQ files in VSEARCH is compliant with the standard as described by Cock et al. (2010) and correctly parses all their tests files. FASTA and FASTQ files are automatically detected and many commands accept both as input. Files compressed with gzip or bzip2 are automatically detected and decompressed using the zlib library by Gailly & Adler (2016) or the bzip2 library by Seward (2016), respectively. Data may also be piped into or out of VSEARCH, allowing for instance many separate FASTA files to be piped into VSEARCH for simultaneous dereplication, or allowing the creation of complex pipelines without ever having to write on slow disks.

VSEARCH is a 64-bit program and allows very large datasets to be processed, essentially limited only by the amount of memory available. The free USEARCH versions are 32-bit programs that limit the available memory to somewhere less than 4GB, often seriously hampering the analysis of realistic datasets.

#### Writing result files

VSEARCH can output results in a variety of formats (FASTA, FASTQ, tables, alignments, SAM) depending on the input format and command used. When outputting FASTA files, the line width may be specified using the *fasta_width* option, where 0 means that line wrapping should be turned off. Similar controls are offered for pairwise or multiple sequence alignments.

#### Searching

Global pairwise sequence comparison is a core functionality of VSEARCH. Several commands compare a query sequence against a database of sequences: all-vs-all alignment (*allpairs_global*), clustering (*cluster_fast*, *cluster_size*, *cluster_smallmem*), chimera detection (*uchime_denovo* and *uchime_ref*) and searching (*usearch_global*). This comparison function proceeds in two phases: an initial heuristic filtering based on shared words, followed by optimal alignment of the query with the most promising candidates.

The first phase is presumably quite similar to USEARCH (Edgar, 2010). Heuristics are used to identify a small set of database sequences that have many words in common with the query sequence. Words (or *k*-mers) consist of a certain number *k* of consecutive nucleotides of a sequence (8 by default, adjustable with the *wordlength* option). All overlapping words are included. A sequence of length *n* then contains at most *n* − *k* + 1 unique words. VSEARCH counts the number of shared words between the query and each database sequence. Words that appear multiple times are counted only once. To count the words in the database sequences quickly, VSEARCH creates an index of all the 4^*k*^ possible distinct words and stores information about which database sequences they appear in. For extremely frequent words, the set of database sequences is represented by a bitmap; otherwise the set is stored as a list. A finer control of *k*-mer indexing is described for USEARCH by the *pattern* (binary string indicating which positions must match) and *slots* options. USEARCH has such options but seems to ignore them. Currently, VSEARCH ignores these two options too. The minimum number of shared words required may be specified with the *minwordmatches* option (10 by default), but a lower value is automatically used for short or simple query sequences with less than 10 unique words.

Comparing sequences based on statistics of shared words is a common method to quickly assess the similarity between two sequences without aligning them, which is often time-consuming. The *D*_2_ statistic and related metrics for alignment-free sequence comparison have often been used for rapid and approximate sequence matching and their statistical properties have been well studied (Song et al., 2014). The approach used here has similarities to the *D*_2_ statistic, but multiple matches of the same word are ignored.

In the second phase, searching proceeds by considering the database sequences in a specific order, starting with the sequence having the largest number of words in common with the query, and proceeding with a decreasing number of shared words. If two database sequences have the same number of words in common with the query, the shortest sequence is considered first. The query sequence is compared with each database sequence by computing the optimal global alignment. The alignment is performed using a multi-threaded and vectorised full dynamic programming algorithm (Needleman & Wunsch, 1970) adapted from SWIPE (Rognes, 2011). Due to the extreme memory requirements of this method when aligning two long sequences, an alternative algorithm described by Hirschberg (1975) and Myers & Miller (1988) is used when the product of the length of the sequences is greater than 25,000,000, corresponding to aligning two 5,000 bp sequences. This alternative algorithm uses only a linear amount of memory but is considerably slower. This second phase is probably where USEARCH and VSEARCH differ the most, as USEARCH by default presumably performs heuristic seed-and-extend alignment similar to BLAST (Altschul et al., 1997), and only performs optimal alignment when the option *fulldp* (full dynamic programming) is used. Computing the optimal pairwise alignment in each case gives more accurate results but is also computationally more demanding. The efficient and vectorised full dynamic programming implementation in VSEARCH compensates that extra cost, at least for sequences that are not too long.

If the resulting alignment indicates a similarity equal to or greater than the value specified with the *id* option, the database sequence is accepted. If the similarity is too low, it is rejected. Several other options may also be used to determine how similarity is computed (*iddef*, as USEARCH used to offer up to version 6), and which sequences should be accepted and rejected, either before (e.g., *self*, *minqsize*) or after alignment (e.g., *maxgaps*, *maxsubs*). The search is terminated when either a certain number of sequences have been accepted (1 by default, adjustable with the *maxaccepts* option), or a certain number of sequences have been rejected (32 by default, adjustable with the *maxrejects* option). The accepted sequences are sorted by sequence similarity and presented as the search results.

VSEARCH also includes a *search_exact* command that only identifies exact matches to the query. It uses a hash table in a way similar to the full-length dereplication command described below.

#### Clustering

VSEARCH includes commands to perform *de novo* clustering using a greedy and heuristic centroid-based algorithm with an adjustable sequence similarity threshold specified with the *id* option (e.g., 0.97). The input sequences are either processed in the user supplied order (*cluster_smallmem*) or pre-sorted based on length (*cluster_fast*) or abundance (the new *cluster_size* option). Each input sequence is then used as a query in a search against an initially empty database of centroid sequences. The query sequence is clustered with the first centroid sequence found with similarity equal to or above the threshold. The search is performed using the heuristic approach described above which generally finds the most similar sequences first. If no matches are found, the query sequence becomes the centroid of a new cluster and is added to the database. If *maxaccepts* is higher than 1, several centroids with sufficient sequence similarity may be found and considered. By default, the query is clustered with the centroid presenting the highest sequence similarity (distance-based greedy clustering, DGC), or, if the *sizeorder* option is turned on, the centroid with the highest abundance (abundance-based greedy clustering, AGC) (He et al., 2015; Westcott & Schloss, 2015; Schloss, 2016). VSEARCH performs multi-threaded clustering by searching the database of centroid sequences with several query sequences in parallel. If there are any non-matching query sequences giving rise to new centroids, the required internal comparisons between the query sequences are subsequently performed to achieve correct results. For each cluster, VSEARCH can create a simple multiple sequence alignment using the center star method (Gusfield, 1993) with the centroid as the center sequence, and then compute a consensus sequence and a sequence profile.

#### Dereplication and rereplication

Full-length dereplication (*derep_fulllength*) is performed using a hash table with an open addressing and linear probing strategy based on the Google CityHash hash functions (written by Geoff Pike and Jyrki Alakuijala, and available at https://github.com/google/cityhash). The hash table is initially empty. For each input sequence, the hash is computed and a lookup in the hash table is performed. If an identical sequence is found, the input sequence is clustered with the matching sequence; otherwise the input sequence is inserted into the hash table.

Prefix dereplication (*derep_prefix*) is also implemented. As with full-length dereplication, identical sequences are clustered. In addition, sequences that are identical to prefixes of other sequences will also be clustered together. If a sequence is identical to the prefix of multiple sequences, it is generally not defined how prefix clustering should behave. VSEARCH resolves this ambiguity by clustering the sequence with the shortest of the candidate sequences. If they are equally long, priority will be given to the most abundant, the one with the lexicographically smaller identifier or the one with the earliest original position, in that order.

To perform prefix dereplication, VSEARCH first creates an initially empty hash table. It then sorts the input sequences by length and identifies the length *s* of the shortest sequence in the dataset. Each input sequence is then processed as follows, starting with the shortest: If an exact match to the full input sequence is found in the hash table, the input sequence is clustered with the matching hash table sequence. If no match to the full input sequence is found, the prefixes of the input sequence are considered, starting with the longest prefix and proceeding with shorter prefixes in order, down to prefixes of length *s*. If a match is now found in the hash table, the sequences are clustered, the matching sequence is deleted from the hash table and the full input sequence is inserted into the hash table instead. If no match is found for any prefix, the full sequence is inserted into the hash table. In the end, the remaining sequences in the hash table will be output with accumulated abundances for all sequences in each cluster.

In order to identify matches in the hash table during prefix dereplication, a hash is computed for each full-length input sequence and all its prefixes. The hash function used is the 64-bit Fowler–Noll–Vo 1a hash function (Fowler, Noll & Vo, 1991), which is simple and quick to compute for such a series of sequences by adding one nucleotide at a time.

The sequences resulting from dereplication and many other commands may be relabeled with a given prefix followed by a sequentially increasing number. VSEARCH exclusively also offers the possibility of relabelling each sequence with the SHA-1 (Eastlake & Jones, 2001) or MD5 (Rivest, 1992) message digest (hash) of the sequence. These are strings that are highly likely to be unique for each sequence. Before the digest is computed, the sequence is normalized by converting U’s to T’s and converting all symbols to upper case. VSEARCH includes public domain code for the MD5 algorithm written by Alexander Peslyak, and for SHA1 by Steve Reid and others.

VSEARCH also includes a new command (*rereplicate*) to perform rereplication that can be used to recreate datasets as they were before full-length dereplication, but of course original labels cannot be recreated.

#### Chimera detection

Chimeras are detected either *de novo* (*uchime_denovo* command) or with a reference database (*uchime_ref* command) using the UCHIME algorithm described by Edgar et al. (2011). VSEARCH will divide each query sequence into four segments and look for similarity of each segment to sequences in the set of potential parents using the heuristic search function described earlier. It will consider the four best candidates for each segment using *maxaccepts* 4 and *maxrejects* 16, and an *id* threshold of 0.55. VSEARCH optionally outputs borderline sequences, that is, sequences having a high enough score (as specified with the *minh* option) but with too small a divergence from the closest parent (as specified with the *mindiv* option). Multi-threading is supported for reference-based chimera detection.

#### Low-complexity sequence masking

VSEARCH includes a highly optimized and parallelized implementation of the Dust algorithm by Tatusov and Lipman for masking of simple repeats and low-complexity nucleotide sequences. It is considerably faster than the implementation of the same algorithm in USEARCH. Their code available at ftp://ftp.ncbi.nlm.nih.gov/pub/tatusov/dust/version1/src/ is in the public domain. VSEARCH uses this algorithm by default, while USEARCH by default uses an undocumented rapid masking algorithm called *fastnucleo*. VSEARCH performs soft-masking automatically for the pairwise alignment, search, clustering and chimera detection commands. This behaviour can be controlled with the *hardmask* option to replace masked symbols with N’s instead of lower-casing them, and the *dbmask* and *qmask* options, which selects the masking algorithm (none, dust or soft) used for the database and query sequences, respectively. Masking may also be performed explicitly on an input file using the *fastx_mask* and *maskfasta* commands.

#### FASTQ file processing

VSEARCH includes commands to detect the FASTQ file version and the range of quality scores used (*fastq_chars*), as well as two commands for computing sequence quality statistics (*fastq_stats* and *fastq_eestats*). It can also truncate and filter sequences in FASTQ files based on various criteria (*fastq_filter*). A new command is added to convert between different FASTQ file versions and quality encodings (*fastq_convert*), e.g., from the old Phred+64 encoded Illumina FASTQ files to the newer Phred+33 format.

#### Merging of paired-end reads

Merging of paired-end reads is supported by VSEARCH using the *fastq_mergepairs* command. The method used has some similarity to PEAR (Zhang et al., 2014) and recognises options similar to USEARCH. The algorithm computes the optimal ungapped alignment of the overlapping region of the forward sequence and the reverse-complemented reverse sequence. The alignment requires a minimum overlap length (specified with the *fastq_minovlen* option, default 10), a maximum number of mismatches (*fastq_maxdiffs* option, default 5), and a minimum and maximum length of the merged sequence (*fastq_minmergelen* option, default 1, and *fastq_maxmergelen* option, default infinite). Staggered read pairs, i.e., read pairs where the 3′ end of the reverse read has an overhang to the left of the 5′  end of the forward read, are not allowed by default, but may be turned on by the *fastq_allowmergestagger* option. VSEARCH uses a match score (alpha) of +4 and a mismatch score (beta) of −5 for perfect quality residues. These scores are weighted by the probability that these two residues really match or mismatch, respectively, taking quality scores into account. These probabilities are computed in a way similar to PEAR score method 2 described in ‘Algorithms and implementation’ of the PEAR paper (Zhang et al., 2014), but VSEARCH assumes all nucleotide background frequencies are 0.25. When merging sequences, VSEARCH computes posterior quality scores for the overlapping regions as described by Edgar & Flyvbjerg (2015). For speed, scores and probabilities are pre-computed for all possible quality scores.

#### Sorting and shuffling

VSEARCH can sort FASTA files by decreasing sequence length (*sortbylength*) or abundance (*sortbysize*). VSEARCH can also perform shuffling of FASTA files in random order (*shuffle*). A seed value for the pseudo random number generator may be provided by the *randseed* option to obtain replicable results.

#### Subsampling

Sequences in FASTA and FASTQ files can be subsampled (*fastx_subsample*) by randomly extracting a certain number (*sample_size*) or percentage (*sample_pct*) of the input sequences. Abundances may be taken into account, giving results as if the input sequences were rereplicated, subsampled and then dereplicated.

## Results and Discussion

### Supported commands and options

VSEARCH implements the following commands available in USEARCH version 7: *allpairs_global*, *cluster_fast*, *cluster_smallmem*, *derep_fulllength*, *derep_prefix*, *fastq_chars*, *fastq_filter*, *fastq_mergepairs*, *fastq_stats*, *fastx_mask*, *maskfasta*, *sortbylength*, *sortbysize*, *uchime_denovo*, *uchime_ref* and *usearch_global*. In addition, the following commands available in USEARCH version 8 have been implemented: *fastq_eestats*, *fastx_revcomp*, *fastx_subsample* and *search_exact*. VSEARCH additionally includes a few new commands that do not exist in USEARCH: *cluster_size*, *fastq_convert*,* rereplicate* and *shuffle*.

Some USEARCH version 7 commands have not yet been implemented in VSEARCH. We have not prioritized commands related to amino acid sequences (*findorfs*), local alignment (*allpairs_local*, *pairs_local*, *search_local*, *ublast*), brute-force search (*search_global*, *pairs_global*), UDB databases (*makeudb_ublast*, *makeudb_usearch*, *udb2fasta*, *udbinfo*, *udbstats*), and the UPARSE pipeline (*cluster_otus*, *uparse_ref*).

Almost all USEARCH 7 options are supported, except for those related to non-standard database indexing (*alpha*, *dbaccelpct*, *dbstep*, *pattern*, *slots*) as well as local alignments and alignment heuristics (*band*, *hspw*, *lext*, *lopen*, *matrix*, *minhsp*, *xdrop_g*, *xdrop_nw*, *xdrop_u*).

The same command and option names as in USEARCH version 7 have generally been used in order to make VSEARCH an almost drop-in replacement. In fact, in QIIME many commands will run fine if an alias or link from usearch to vsearch is made. Detailed documentation of VSEARCH is available as a man page. We will consider adding further commands and options to VSEARCH in the future.

### Performance assessment

The performance of the most important functions of VSEARCH version 2.0.3 (64-bit) was evaluated and compared to the 32-bit versions of USEARCH version 7.0.1090 and 8.1.1861. All datasets used were small enough to fit comfortably in the memory allocated to a 32-bit process. Chimera detection was also compared to UCHIME version 4.2. All tests were run on GNU/Linux CentOS 6.7 compute nodes with 16 physical cores (Intel(R) Xeon(R) CPU E5-2670 0 @ 2.60 GHz) and 64GB RAM. Programs were run with 8 threads, if possible. All times indicated are wall-clock times. All scripts and data necessary to perform the evaluations are available in the GitHub repository at https://github.com/torognes/vsearch-eval/ to enable independent replication.

#### Searching

**Figure 1:**
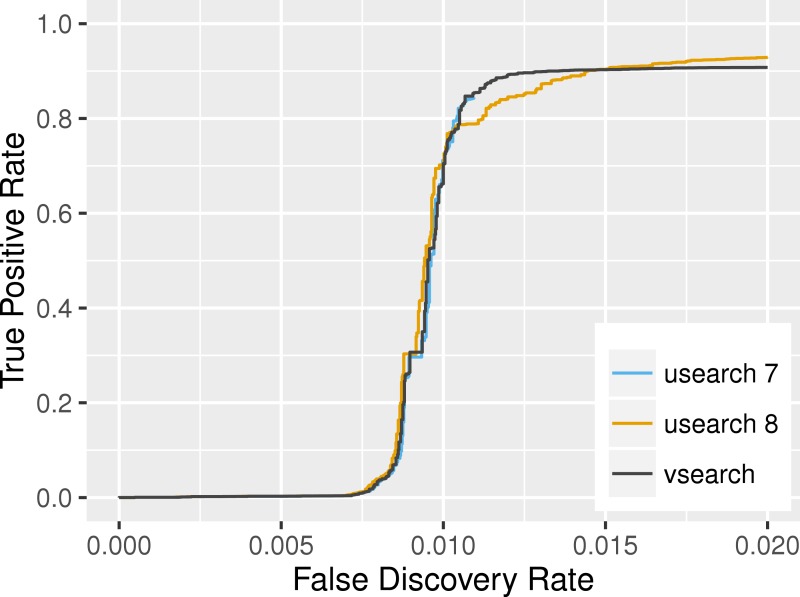
Search accuracy on the RFAM v11 dataset. USEARCH version 7 (blue), USEARCH version 8 (orange) and VSEARCH (black) was run using the *usearch_global* command on subsets of the RFAM dataset to identify members of the same families. The plot shows the true positive rate (also known as the recall or sensitivity) as a function of the false discovery rate at varying sequence similarity levels. This curve is based on data from 20 shufflings of the dataset.

Evaluation of search accuracy was carried out as described in the USEARCH paper (Edgar, 2010), its supplementary, and on the website (http://drive5.com/usearch/benchmark_rfam.html), by assessing the ability of the programs to identify RNA sequences belonging to the same family in RFAM (Burge et al., 2013). The 383,004 sequences in Rfam version 11 were randomly shuffled and then the first sequence from each of the 2,085 (out of 2,208) families that contained at least 2 members was selected as a representative and used as a query against the remaining 380,919 sequences. The programs were run with options *id* 0.0, *minseqlength* 1, *maxaccepts* 1, *maxrejects* 32, and *strand* plus. If the matching sequence found belonged to the same family, it was considered a true positive, otherwise it was considered as a false positive. We combined the results from 20 shufflings and plotted the results in the ROC-like curve shown in Fig. 1. For a false discovery rate between 0.010 and 0.015, VSEARCH is more accurate than USEARCH’s latest version. For lower values, the three programs have similar accuracies. At higher false discovery rates, USEARCH version 8 has an advantage.

The time to search the Rfam database as described above was measured. To avoid extremely short running times, 1,000 replicates of the datasets were used. USEARCH version 7 required on average 5 min 29 s for the search, USEARCH version 8 took 5 min 57 s, while VSEARCH took 5 min 26 s.

#### Clustering

Westcott & Schloss (2015) have already carried out an evaluation of the clustering performance of VSEARCH. They tested the ability of several tools to assign OTUs for 16S rRNA sequences and “demonstrated that for the greedy algorithms VSEARCH produced assignments that were comparable to those produced by USEARCH making VSEARCH a viable free and open source alternative to USEARCH”. Schloss (2016) also evaluated *de novo* clustering by VSEARCH.

**Figure 2:**
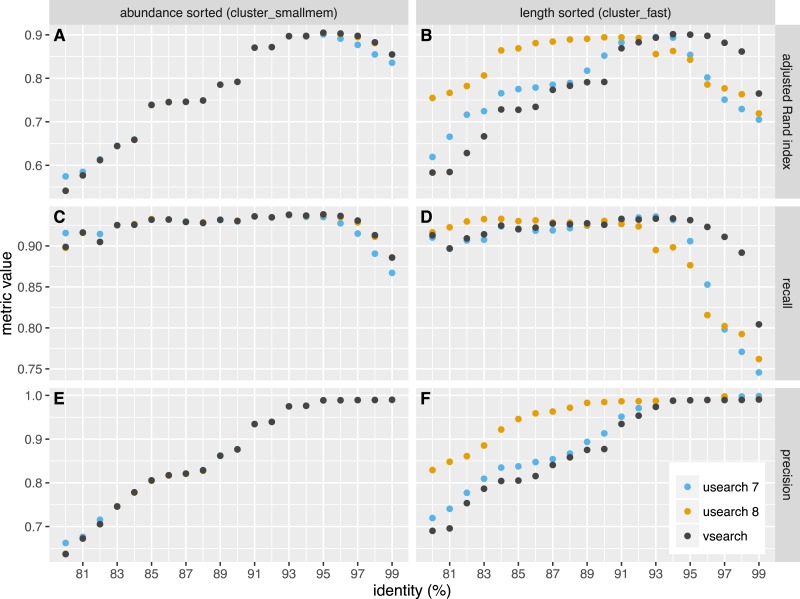
Clustering accuracy on the even dataset. USEARCH version 7 (blue) and 8 (orange) and VSEARCH (black) was run using abundance sorting (*cluster_smallmem*) (A, C, E) and length sorting (*cluster_fast*) (B, D, F) on the even dataset. The performance is indicated with the adjusted Rand index (A, B), recall (C, D) and precision (E, F) metrics.

**Figure 3:**
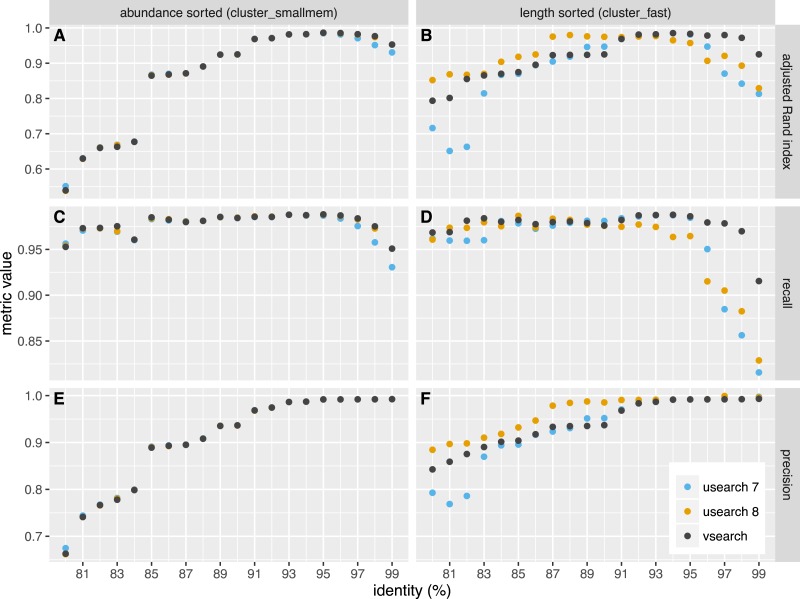
Clustering accuracy on the uneven dataset. USEARCH version 7 (blue) and 8 (orange) and VSEARCH (black) was run using abundance sorting (*cluster_smallmem*) (A, C, E) and length sorting (*cluster_fast*) (B, D, F) on the uneven dataset. The performance is indicated with the adjusted Rand index (A, B), recall (C, D) and precision (E, F) metrics.

We independently evaluated the clustering accuracy of USEARCH and VSEARCH as described for Swarm (Mahé et al., 2014) using two mock datasets, one with an even and one with uneven composition of 57 archaea and bacteria. The datasets were first dereplicated. Then the taxonomy of the unique sequences was assigned by a search against the set of rRNA reference sequences representing the species in the mock datasets, carried out with the *usearch_global* command of USEARCH. The sequences were shuffled randomly 10 times and clustering was performed at 20 different similarity levels ranging from 80% to 99% in steps of 1%. Clustering was carried out in two ways, first using the *cluster_fast* command that pre-sorts the sequences by length, and then using the *cluster_smallmem* command that simply processes the sequences in the user-supplied order (in that case, we supplied sequences ordered by decreasing abundance using the *sortbysize* command). We then compared the clusters obtained to the assigned species and computed the recall, precision and the adjusted Rand index of the classifications. Recall measures to what extent amplicons assigned to the same species are grouped together in the same OTU (i.e., not over-splitting). Precision measures to what extent amplicons in an OTU are assigned to the same species (i.e., not over-grouping). The adjusted Rand index (Rand, 1971; Hubert & Arabie, 1985) summarises both precision and recall using a measure of agreement between two clusterings while adjusting for expected values by chance. The average values over the all shufflings are presented in Figs. 2 and 3 for the even and uneven datasets, respectively. For abundance-sorted sequences, the difference between VSEARCH and USEARCH version 8 is negligible. The difference is larger for length-sorted sequences. When using length sorting, USEARCH 8 (as well as version 7 on the even dataset) shows better precision than VSEARCH for similarity levels below 93%. However, OTU delineation is usually performed at higher similarity values, typically 97%. In the case of our benchmark, overall accuracy as measured by the adjusted Rand index is maximised at 95–97% similarity. This is precisely the region where for length sorting at least VSEARCH outperforms USEARCH.

**Figure 4:**
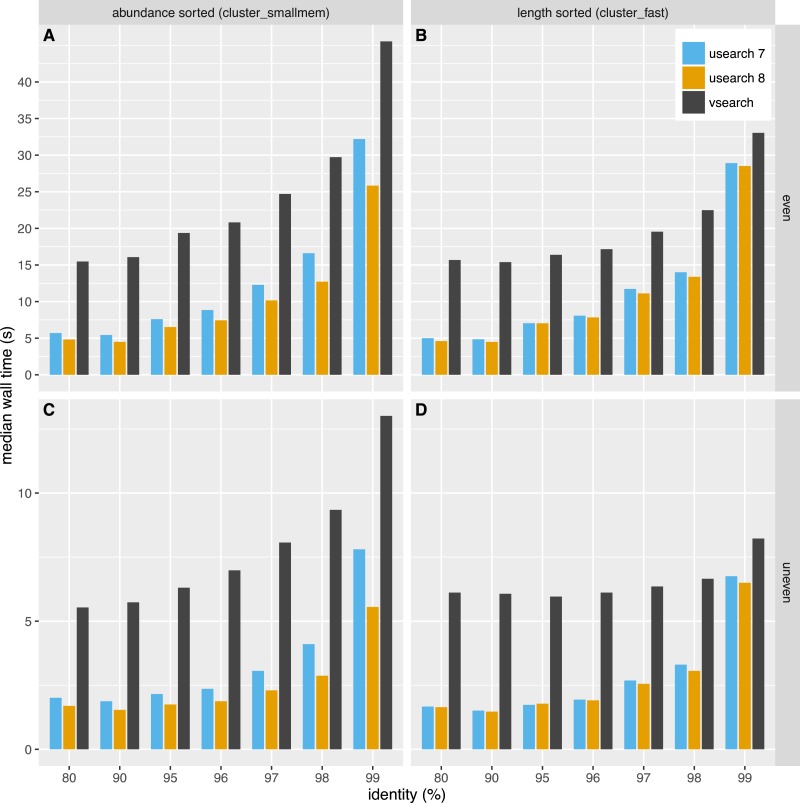
Clustering speed. Median wall time in seconds to cluster the even (A, B) and uneven (C, D) datasets using USEARCH version 7 (blue) and 8 (orange) and VSEARCH (black) using abundance sorting (*cluster_smallmem*) (A, C) and length sorting (*cluster_fast*) (B, D).

The time used for clustering is shown in Fig. 4. The time used depended on the dataset, algorithm and clustering threshold. The USEARCH programs were in general 2–3 times faster than VSEARCH. In general the difference in speed was smaller for higher thresholds, especially at 99% similarity. Clustering with USEARCH using the *fulldp* option is an order of magnitude slower than VSEARCH, but contrary to our expectations it does not seem to improve accuracy.

#### Dereplication

Measurements of dereplication speed were performed on the even and uneven datasets described earlier as well as on the BioMarKs dataset (Logares et al., 2014). For full-length dereplication (*derep_fulllength*) VSEARCH was about 40–50% faster than USEARCH version 7 and 50–70% faster than version 8 on all three datasets. All programs were approximately equally fast on prefix dereplication (*derep_prefix*) of the even and uneven datasets. However, prefix dereplication of the BioMarKs dataset was extremely slow with USEARCH. USEARCH version 7 used more than 4 min and version 8 more than 27 min, while VSEARCH used less than 4 s. The prefix dereplication algorithm used in USEARCH appears ineffective when dealing with short sequences. Removing the 811 sequences shorter than 200 bp out of the 312,503 sequences of the BioMarKs dataset reduces the running time of USEARCH version 7 and 8 down to just 5 and 6 s, respectively.

#### Chimera detection

We evaluated the chimera detection accuracy of VSEARCH and USEARCH in two ways, first using a method similar to that performed for UCHIME, and then using a new chimera simulation procedure based on sequences from Greengenes (DeSantis et al., 2006) and SILVA (Quast et al., 2013) sequences.

**Table 1:** Chimera detection performance with the SIMM dataset. UCHIME (UC), USEARCH version 7 (U7) and 8 (U8), and VSEARCH (V) was run using the *uchime_ref* algorithm on the SIMM dataset that was originally also used to evaluate the UCHIME algorithm. Divergence is the percentage of similarity to the original sequences. Noise is either zero (–) or the percentage of indels (i1–i5) or substitutions (m1-5) added. The number of chimeras detected out of 100 of each type is shown. The best results in each category are shaded.

		2 segments				3 segments				4 segments			
Divergence	Noise	UC	U7	U8	V	UC	U7	U8	V	UC	U7	U8	V
**97–99%**	**–**	89	88	88	89	56	52	52	55	38	33	34	35
	**i1**	79	79	77	85	46	44	43	53	32	27	24	34
	**i2**	64	57	56	77	33	32	31	56	24	20	18	33
	**i3**	48	45	36	72	37	35	29	45	16	17	16	21
	**i4**	29	24	23	65	18	11	13	40	9	9	8	25
	**i5**	27	22	16	53	15	12	12	39	7	8	6	17
	**m1**	83	83	83	81	53	48	48	53	33	29	29	30
	**m2**	73	71	71	72	49	44	44	50	28	22	22	27
	**m3**	66	66	66	68	40	40	39	44	21	20	21	21
	**m4**	55	54	53	57	28	24	23	28	21	18	18	19
	**m5**	44	44	42	48	20	19	18	28	16	14	12	12
**95–97%**	**–**	100	100	100	100	80	77	76	79	64	60	59	63
	**i1**	100	98	98	100	77	75	72	75	54	55	53	61
	**i2**	96	94	93	99	60	55	55	71	48	44	44	60
	**i3**	86	82	82	95	61	50	52	70	38	36	31	53
	**i4**	75	66	64	95	48	41	39	64	29	29	22	47
	**i5**	64	58	53	86	37	32	25	60	24	19	19	46
	**m1**	99	99	99	99	76	73	73	76	60	57	57	60
	**m2**	98	97	97	97	71	69	69	71	50	48	46	48
	**m3**	93	94	94	96	63	61	61	64	41	41	41	42
	**m4**	92	92	90	93	56	55	54	57	39	39	37	41
	**m5**	86	86	85	86	53	51	51	56	35	35	34	34
**90–95%**	**–**	100	100	100	100	93	93	93	93	88	88	88	86
	**i1**	100	100	100	100	88	88	87	91	86	86	87	88
	**i2**	99	97	99	99	83	79	78	88	74	72	72	84
	**i3**	100	100	100	100	79	76	75	88	74	69	70	82
	**i4**	99	94	96	99	80	71	72	84	66	62	61	79
	**i5**	95	84	86	99	74	65	65	88	55	48	48	71
	**m1**	100	100	100	100	89	89	89	92	87	87	86	85
	**m2**	100	100	100	100	87	87	87	89	78	78	78	79
	**m3**	100	99	99	100	86	86	86	89	76	76	78	80
	**m4**	100	100	100	100	82	82	84	83	73	73	72	78
	**m5**	99	98	98	99	82	81	82	84	75	73	75	79

First we repeated the evaluation of the *uchime_ref* command described in the UCHIME paper (Edgar et al., 2011) using the SIMM dataset downloaded from http://drive5.com/uchime/uchime_download.html. The dataset consists of 900 simulated chimeras that are approximately 250 bp long. The chimeras were generated from 2, 3 or 4 segments selected randomly from 86 original sequences and have similarities in the ranges 90–95%, 95–97% and 97–99% to the original sequences. They were either used unmodified or with 1–5% indels or 1–5% substitutions. We assessed the performance of (i) the original open-source UCHIME version 4.2 program, (ii) USEARCH version 7, (iii) USEARCH version 8, and (iv) VSEARCH. The results are shown in Table 1 and indicate that VSEARCH is superior to the other tools in almost all cases, and in particular when indels were added. The original UCHIME program was found to be quite, but also considerably slower than all the other tools. USEARCH was better than VSEARCH in only 4 out of 99 cases.

**Figure 5:**
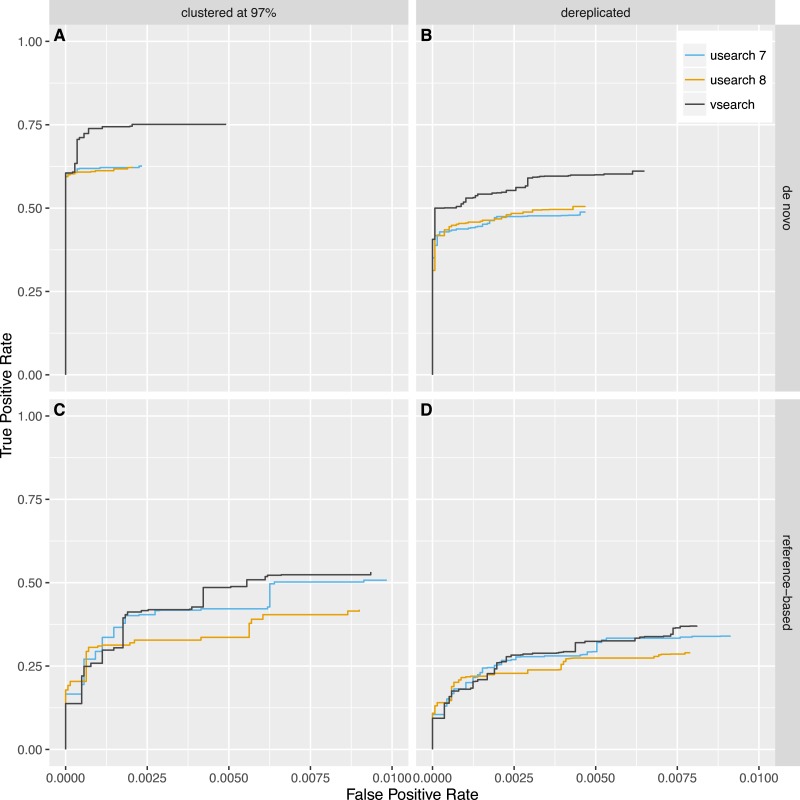
Chimera detection performance with the Greengenes dataset shown with ROC curves. USEARCH version 7 (blue) and 8 (orange) and VSEARCH (black) was run using the *uchime_denovo* (A, B) and the *uchime_ref* (C, D) commands on simulated Illumina data based on the Greengenes database that has either been clustered with a 97% identity threshold (using the *cluster_fast* command in VSEARCH) (A, C) or dereplicated (using the *derep_fulllength* command in VSEARCH) (B, D).

**Figure 6:**
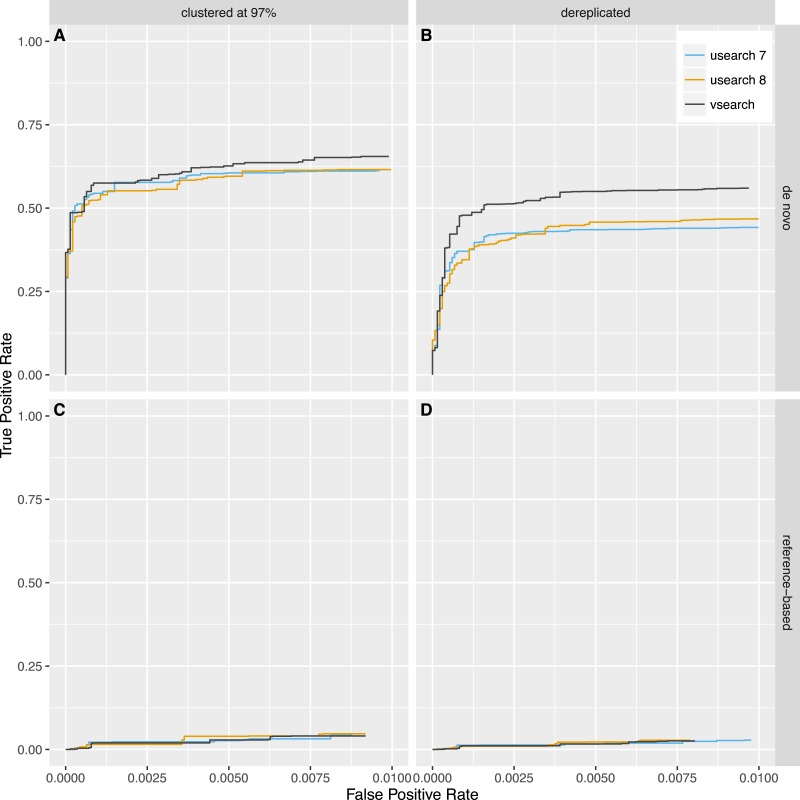
Chimera detection performance on the SILVA dataset shown with ROC curves. USEARCH version 7 (blue) and 8 (orange) and VSEARCH (black) was run using the *uchime_denovo* (A, B) and the *uchime_ref* (C, D) commands on simulated Illumina data based on the SILVA database that has either been clustered with a 97% identity threshold (using the *cluster_fast* command in USEARCH) (A, C) or dereplicated (using the *derep_fulllength* command in VSEARCH) (B, D).

Next, we tested reference-based (*uchime_ref*) and *de novo* (*uchime_denovo*) chimera detection using sequences from the 2011 version of Greengenes downloaded from http://greengenes.lbl.gov/Download/Sequence_Data/Fasta_data_files/ and from version 106 (May 2011) of the SILVA database downloaded from https://www.arb-silva.de/no_cache/download/archive/release_106/Exports/. Sequences from the 16S rRNA V4 region was computationally extracted using the 515F (5′-GTGNCAGCMGCCGCGGTAA-3′) and 806R (5′-GGACTACHVGGGTWTCTAAT-3′) primers, and 8,000 reads were randomly selected from each database. PCR was simulated using a new simulation algorithm known as Simera (Nichols & Quince, 2016) (available at https://github.com/bnichols1979/Simera) that includes amplification and creation of PCR artefacts like chimeras. We sampled 30,000 reads (-s 30,000) and generated 20,000 potential chimeras (-c 20,000). Defaults were used for other options to Simera. The output sequences were then fed into an Illumina MiSeq noise simulator (Schirmer et al., 2015) ending up with 14,966 reads based on Greengenes and 14,952 reads based on SILVA, of which 1,262 and 1,640 reads contain chimeric sequences, respectively. Next, the sequences were either clustered using the *cluster_fast* command at 97% identity or dereplicated. VSEARCH and USEARCH version 7 and 8 were run using the *uchime_denovo* command and then using the *uchime_ref* command with the Gold database downloaded from http://drive5.com/uchime/uchime_download.html as the reference database. To assess the performance, the results were sorted based on the chimera score, and then the ability to classify individual sequences correctly into chimeric and non-chimeric was plotted as ROC curves. The curves reflect the accuracy of classifying individual reads, not clusters, as abundances were taken into account. The plots in Figs. 5 and 6 show that *de novo* chimera detection performs better than reference-based detection, with the SILVA dataset in particular, but it does of course depend on the reference database used. VSEARCH performs better than both versions of USEARCH for *de novo* chimera detection. For reference-based detection VSEARCH also performs better for the Greengenes dataset, while none of the programs work well with the SILVA dataset. Clustering at 97% appears to be more appropriate than dereplication. In this test, the USEARCH programs were about twice as fast as VSEARCH for *de novo* detection, while they were about 10–30% faster than VSEARCH for reference-based detection.

### Merging of paired-end reads

Evaluation of paired-end reads merging performance was carried out in a manner similar to that described for the evaluation of PEAR (Zhang et al., 2014). We used whole genome sequencing data from *Staphylococcus aureus* subspecies aureus strain USA 300 TCH 1516 sequenced by MacCallum et al. (2009) and retrieved from the GAGE-B repository (http://ccb.jhu.edu/gage_b/). The *S.aureus* reads were 101 bp long from on average 180 bp long fragments, giving a 45X coverage of the genome. We also used *Methylococcus capsulatus* strain Bath 16S rRNA V3 region amplicon reads sequenced by Masella et al. (2012). These reads were 108 bp long and the pairs should have an overlap of exactly 18 bp. Merging options were set to allow a minimum overlap of 10 bp and a maximum of 5 mismatches (USEARCH 7 and 8 have different default values for those), while other options were left at defaults. All programs were run with 8 threads. Merged sequences that could be perfectly aligned to their respective reference sequences (either the entire genome or the specific rRNA region) using BWA MEM (Li & Durbin, 2009) were considered correctly merged. The results are shown in Table 2. The numbers indicate that USEARCH version 7 merges the most reads for both bacteria, but also has the lowest percentage of correctly merged pairs of those merged. USEARCH version 8 merges the fewest reads, but has the highest percentage of correctly merged reads of those merged. VSEARCH is in the middle by merging more reads than USEARCH 8 with only a small decrease in the percentage of correct merges. VSEARCH is about twice as fast as USEARCH 8 and 4–5 times faster than USEARCH version 7.

**Table 2:** Paired-end reads merging performance. The number of sequence pairs, merged pairs, and correctly merged pairs are shown for each bacterium and program. The percentage of reads merged, as well as the percentage of correctly merged reads both of the merged reads and of all reads are also shown. Times are in seconds using 8 threads.

Bacterium	Program	Pairs	Merged	Correct	%Merged	%Cor/Mer	%Cor/All	Time (s)
*Staphylococcus aureus*	USEARCH 7	647,052	273,438	270,849	42.26	99.05	41.86	11.65
	USEARCH 8	647,052	203,729	202,003	31.49	99.15	31.22	4.69
	VSEARCH	647,052	214,988	213,103	33.23	99.12	32.93	2.15
*Methylococcus capsulatus* strain Bath	USEARCH 7	673,845	643,903	642,720	95.56	99.82	95.38	14.43
	USEARCH 8	673,845	554,099	553,747	82.23	99.94	82.18	6.27
	VSEARCH	673,845	581,752	581,346	86.33	99.93	86.27	3.61

**Figure 7:**
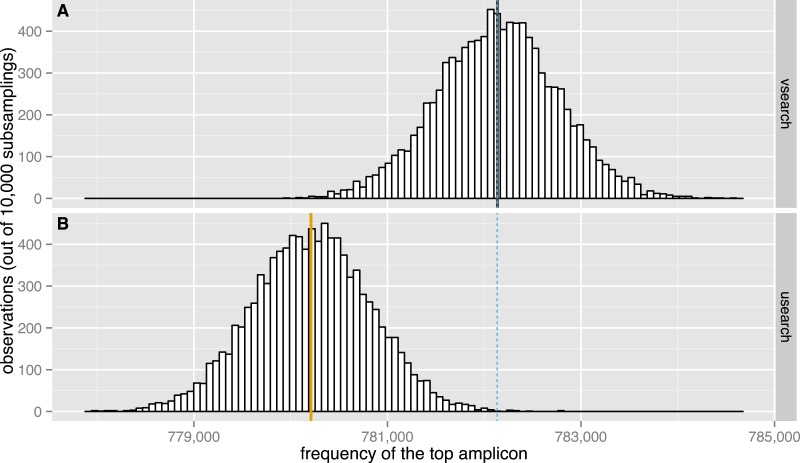
Subsampling performance. The observed distribution of the maximum amplicon abundance in 10,000 random subsamplings of 5% of the TARA V9 dataset using VSEARCH (A, black) and USEARCH version 8 (B, orange) is shown. The expected mean abundance is 782,133.5 (blue dashed line).

#### Subsampling

We evaluated the subsampling commands of USEARCH version 8 and VSEARCH to check if the results obtained correspond to those expected. We performed 10,000 random subsamplings of 5% of the 9.5 million unique sequences in the TARA V9 dataset (Karsenti et al., 2011). To make this possible with the 32-bit USEARCH, we first downsampled the dataset once to 10% using VSEARCH and then randomly subsampled it again at 50% with either USEARCH or VSEARCH. Plots of the distribution of the abundance of the most abundant sequence in each subsampling are shown in Fig. 7. The highest amplicon abundance in the original dataset is 15,638,316. After the initial 10% subsampling, the highest abundance was 1,564,267. After the second subsampling, the top abundances should therefore have a distribution centred on a value of 782,133.5. As can be seen from the figure, the USEARCH distribution has a mean that is about 2,000 too small, while the VSEARCH distribution is correctly centred on the expected value. Subsampling experiments were also performed at 2.5%, 1.5% and 0.5% with similar results, although the errors were of decreasing size. USEARCH seems to under-sample abundant amplicons and to over-sample rare amplicons.

## Conclusions

VSEARCH supports almost all of the commands and options for nucleotide sequence analysis in USEARCH version 7 as well as several new features. It has a 64-bit design and handles large datasets virtually only limited by the amount of available memory. We have demonstrated that VSEARCH is in general more accurate than USEARCH when performing searching, clustering, chimera detection and subsampling. The accuracy is on a par with USEARCH for paired-end reads merging. VSEARCH is faster than USEARCH when performing dereplication and merging of paired-end reads, but slower for clustering and chimera detection. We will continue to improve the accuracy, speed and robustness of VSEARCH in the future, as well as adding new features.

## Additional Information and Declarations

### Data Availability

The following information was supplied regarding data availability: VSEARCH is freely available at https://github.com/torognes/vsearch under a dual license, either the GNU General Public License version 3, or the BSD 2-clause license. Binaries are provided for x86-64 systems running GNU/Linux or OS X (10.7 or higher). The repository with scripts and data for the evaluation is available at https://github.com/torognes/vsearch-eval. Thanks to the work of several people, there is now a vsearch package in Debian and a vsearch package for Homebrew, as well as a Galaxy wrapper for VSEARCH in the Galaxy ToolShed.

## References

[ref-1] Altschul SF, Madden TL, Schäffer AA, Zhang J, Zhang Z, Miller W, Lipman DJ (1997). Gapped BLAST and PSI-BLAST: a new generation of protein database search programs. Nucleic Acids Research.

[ref-2] Burge SW, Daub J, Eberhardt R, Tate J, Barquist L, Nawrocki EP, Eddy SR, Gardner PP, Bateman A (2013). Rfam 11.0: 10 years of RNA families. Nucleic Acids Research.

[ref-3] Caporaso JG, Kuczynski J, Stombaugh J, Bittinger K, Bushman FD, Costello EK, Fierer N, Peña AG, Goodrich JK, Gordon JI, Huttley GA, Kelley ST, Knights D, Koenig JE, Ley RE, Lozupone CA, Mcdonald D, Muegge BD, Pirrung M, Reeder J, Sevinsky JR, Turnbaugh PJ, Walters WA, Widmann J, Yatsunenko T, Zaneveld J, Knight R (2010). QIIME allows analysis of high-throughput community sequencing data. Nature Methods.

[ref-4] Cock PJA, Fields CJ, Goto N, Heuer ML, Rice PM (2010). The Sanger FASTQ file format for sequences with quality scores, and the Solexa/Illumina FASTQ variants. Nucleic Acids Research.

[ref-5] DeSantis TZ, Hugenholtz P, Larsen N, Rojas M, Brodie EL, Keller K, Huber T, Dalevi D, Hu P, Andersen GL (2006). Greengenes, a chimera-checked 16S rRNA gene database and workbench compatible with ARB. Applied and Environmental Microbiology.

[ref-6] Eastlake D, Jones P (2001). US Secure Hash Algorithm 1 (SHA). ftp://ftp.rfc-editor.org/in-notes/rfc3174.txt.

[ref-7] Edgar RC (2010). Search and clustering orders of magnitude faster than BLAST. Bioinformatics.

[ref-8] Edgar RC (2013). UPARSE: highly accurate OTU sequences from microbial amplicon reads. Nature Methods.

[ref-9] Edgar RC, Flyvbjerg H (2015). Error filtering, pair assembly and error correction for next-generation sequencing reads. Bioinformatics.

[ref-10] Edgar RC, Haas BJ, Clemente JC, Quince C, Knight R (2011). UCHIME improves sensitivity and speed of chimera detection. Bioinformatics.

[ref-11] Fowler G, Noll LC, Vo P (1991). Fowler / Noll / Vo (FNV) hash. http://www.isthe.com/chongo/tech/comp/fnv/index.html.

[ref-12] Gailly JL, Adler M (2016). zlib: a massively spiffy yet delicately unobtrusive compression library. http://www.zlib.net/.

[ref-13] Gilbert JA, Jansson JK, Knight R (2014). The Earth Microbiome project: successes and aspirations. BMC Biology.

[ref-14] Gusfield D (1993). Efficient methods for multiple sequence alignment with guaranteed error bounds. Bulletin of Mathematical Biology.

[ref-15] He Y, Caporaso JG, Jiang XT, Sheng HF, Huse SM, Rideout JR, Edgar RC, Kopylova E, Walters WA, Knight R, Zhou HW (2015). Stability of operational taxonomic units: an important but neglected property for analyzing microbial diversity. Microbiome.

[ref-16] Hirschberg DS (1975). A linear space algorithm for computing maximal common subsequences. Communications of the ACM.

[ref-17] Hubert L, Arabie P (1985). Comparing partitions. Journal of Classification.

[ref-18] Human Microbiome Project Consortium (2012). Structure, function and diversity of the healthy human microbiome. Nature.

[ref-19] Karsenti E, González Acinas S, Bork P, Bowler C, De Vargas C, Raes J, Sullivan MB, Arendt D, Benzoni F, Claverie J-M, Follows M, Jaillon O, Gorsky G, Hingamp P, Iudicone D, Kandels-Lewis S, Krzic U, Not F, Ogata H, Pesant S, Reynaud EG, Sardet C, Sieracki ME, Speich S, Velayoudon D, Weissenbach J, Wincker P, the Tara Oceans Consortium (2011). A holistic approach to marine eco-systems biology. PLoS Biology.

[ref-20] Li H, Durbin R (2009). Fast and accurate short read alignment with Burrows-Wheeler transform. Bioinformatics.

[ref-21] Logares R, Audic S, Bass D, Bittner L, Boutte C, Christen R, Claverie J-M, Decelle J, Dolan JR, Dunthorn M, Edvardsen B, Gobet A, Kooistra WHCF, Mahé F, Not F, Ogata H, Pawlowski J, Pernice MC, Romac S, Shalchian-Tabrizi K, Simon N, Stoeck T, Santini S, Siano R, Wincker P, Zingone A, Richards T, De Vargas C, Massana R (2014). The patterning of rare and abundant community assemblages in coastal marine-planktonic microbial eukaryotes. Current Biology.

[ref-22] MacCallum I, Przybylski D, Gnerre S, Burton J, Shlyakhter I, Gnirke A, Malek J, McKernan K, Ranade S, Shea TP, Williams L, Young S, Nusbaum C, Jaffe DB (2009). ALLPATHS 2: small genomes assembled accurately and with high continuity from short paired reads. Genome Biology.

[ref-23] Mahé F, Rognes T, Quince C, De Vargas C, Dunthorn M (2014). Swarm: robust and fast clustering method for amplicon-based studies. PeerJ.

[ref-24] Masella AP, Bartram AK, Truszkowski JM, Brown DG, Neufeld JD (2012). PANDAseq: paired-end assembler for illumina sequences. BMC Bioinformatics.

[ref-25] Myers EW, Miller W (1988). Optimal alignments in linear space. Computer Applications in the Biosciences.

[ref-26] Needleman SB, Wunsch CD (1970). A general method applicable to the search for similarities in the amino acid sequence of two proteins. Journal of Molecular Biology.

[ref-27] Nichols B, Quince C (2016). Simera: Modelling the PCR Process to Simulate Realistic Chimera Formation. bioRxiv.

[ref-28] Quast C, Pruesse E, Yilmaz P, Gerken J, Schweer T, Yarza P, Peplies J, Glöckner FO (2013). The SILVA ribosomal RNA gene database project: improved data processing and web-based tools. Nucleic Acids Research.

[ref-29] Rand WM (1971). Objective criteria for the evaluation of clustering methods. Journal of the American Statistical Association.

[ref-30] Rivest R (1992). The MD5 message-digest algorithm. ftp://ftp.rfc-editor.org/in-notes/rfc1321.txt.

[ref-31] Rockström J, Steffen W, Noone K, Persson A, Chapin 3rd FS, Lambin EF, Lenton TM, Scheffer M, Folke C, Schellnhuber HJ, Nykvist B, De Wit CA, Hughes T, Van der Leeuw S, Rodhe H, Sörlin S, Snyder PK, Costanza R, Svedin U, Falkenmark M, Karlberg L, Corell RW, Fabry VJ, Hansen J, Walker B, Liverman D, Richardson K, Crutzen P, Foley JA (2009). A safe operating space for humanity. Nature.

[ref-32] Rognes T (2011). Faster Smith-Waterman database searches by inter-sequence SIMD parallelisation. BMC Bioinformatics.

[ref-33] Schirmer M, Ijaz UZ, D’Amore R, Hall N, Sloan WT, Quince C (2015). Insight into biases and sequencing errors for amplicon sequencing with the Illumina MiSeq platform. Nucleic Acids Research.

[ref-34] Schloss PD (2016). Application of a database-independent approach to assess the quality of operational taxonomic unit picking methods. mSystems.

[ref-35] Schloss PD, Westcott SL, Ryabin T, Hall JR, Hartmann M, Hollister EB, Lesniewski RA, Oakley BB, Parks DH, Robinson CJ, Sahl JW, Stres B, Thallinger GG, Van Horn DJ, Weber CF (2009). Introducing mothur: open-source, platform-independent, community-supported software for describing and comparing microbial communities. Applied and Environmental Microbiology.

[ref-36] Seward J (2016). bzip2 and libbzip2. http://www.bzip.org/.

[ref-37] Song K, Ren J, Reinert G, Deng M, Waterman MS, Sun F (2014). New developments of alignment-free sequence comparison: measures, statistics and next-generation sequencing. Briefings in Bioinformatics.

[ref-38] Steffen W, Richardson K, Rockström J, Cornell SE, Fetzer I, Bennett EM, Biggs R, Carpenter SR, De Vries W, De Wit CA, Folke C, Gerten D, Heinke J, Mace GM, Persson LM, Ramanathan V, Reyers B, Sörlin S (2015). Sustainability. Planetary boundaries: guiding human development on a changing planet. Science.

[ref-39] Westcott SL, Schloss PD (2015). *De novo* clustering methods outperform reference-based methods for assigning 16S rRNA gene sequences to operational taxonomic units. PeerJ.

[ref-40] Zhang J, Kobert K, Flouri T, Stamatakis A (2014). PEAR: a fast and accurate Illumina Paired-End reAd mergeR. Bioinformatics.

